# Zopiclone-Induced Methemoglobinemia: A Case Report Highlighting How the Treatment Differs in Patients on Serotonergic Medication

**DOI:** 10.7759/cureus.54224

**Published:** 2024-02-15

**Authors:** Lionel Kee Yon See, Grace Min Yi Fong, Dorcas Lim

**Affiliations:** 1 General Medicine, Sengkang General Hospital, Singapore, SGP

**Keywords:** depression, methylene blue, ascorbic acid, acquired methemoglobinemia, overdose, zopiclone

## Abstract

Zopiclone is a sedative-hypnotic that is increasingly being used for insomnia, especially among patients with depression. The side effects of zopiclone include nausea, vomiting, headache, giddiness, sedation, altered mental status, and coma. Here, we describe a rare case of a patient with underlying depression who overdosed on zopiclone, resulting in a presentation of drowsiness and dyspnea. A diagnosis of methemoglobinemia was made only through astute observation of the presence of a saturation gap, poor oxygen saturation despite high flow oxygen supplementation, and the arterial blood gas sample appearing chocolate brown in color. Treatment of such patients usually includes the gold standard of methylene blue. However, in our case, there was a risk of serotonin syndrome as the patient was on a serotonergic antidepressant prior. As such, an alternative treatment with ascorbic acid was utilized instead. Methemoglobinemia, while uncommon, should always be suspected in patients who present with zopiclone overdose as it can be life-threatening and is easily treatable.

## Introduction

Methemoglobinemia can be congenital or acquired, where ferrous heme iron (Fe^2+^) is oxidised to ferric heme iron (Fe^3+^), impairing oxygen binding. Furthermore, the ferric heme in the hemoglobin tetramer causes the remaining normal ferrous heme within the same tetramer to have increased oxygen affinity, thereby shifting the oxygen dissociation curve to the left, reducing oxygen delivery to tissues [1]. As such, patients with severe methemoglobinemia can die from hypoxia despite administration of high levels of supplemental oxygen. The vast majority of cases of methemoglobinemia are acquired. This can range from exposure to drugs such as dapsone [2], topical anesthetic drugs [3,4] or antimalarial medications [5], as well as from certain foods [6] or chemical preservatives such as those with high levels of nitrates or nitrites [7], among others.

Overdose with zopiclone is usually associated with nausea or vomiting, giddiness, confusion, lethargy, drowsiness, and in the worst case, coma. It is not expected to cause methemoglobinemia. Here, we describe a rare case of zopiclone-induced methemoglobinemia in a patient on anti-depressant medication. Treatment with the gold standard of methylene blue was not possible, and hence, alternative forms of treatment had to be utilized.

## Case presentation

A 78-year-old female with a past medical history of hypertension, hyperlipidemia, depression with insomnia, and recently diagnosed systemic lupus erythematosus (SLE) with lupus nephritis on prednisolone presents with drowsiness. Her son had found her at home in a semi-conscious state, unable to open her eyes. She was still able to move her upper limbs weakly but was unable to move her lower limbs.

He immediately called for the ambulance, and in the emergency department, it was noted that the patient’s SpO_2_ on the pulse oximeter fluctuated between 90-92% even when on 100% oxygen via face mask. She appeared slightly dyspneic as well with a respiratory rate of 24 breaths per minute and increased accessory muscle use. Her arterial blood gas sample was noted to be chocolate brown in color, and results showed pH 7.38, PaO_2_ 125mmHg, PaCO_2_ 37mmHg, and SaO_2_ 100%. She was subsequently placed on a high-flow nasal cannula, with 60L/min of oxygen. A repeat arterial blood gas showed persistence of the saturation gap, with PaO_2_ 278mmHg and SaO_2_ 100%, but a SpO_2_ of 91%. Methemoglobin results came back elevated at 10.4%.

Other blood investigations are shown in Table 1. Importantly, her hemoglobin levels were stable at 8.2g/dL. There was no evidence of hemolysis given by the normal bilirubin, haptoglobin, and lactate dehydrogenase levels. She had kidney impairment from her underlying lupus nephritis, but this was stable from two months prior. Iron studies showed anemia of chronic disease with raised ferritin levels that were also stable from two months prior. Her chest X-ray was otherwise clear and there were no obvious sources of infection. A computed tomography (CT) scan of her brain to evaluate for drowsiness was normal. She did not have glucose-6-phosphate-dehydrogenase (G6PD) deficiency.

**Table 1:** Initial blood investigations

Investigations		Value	Normal values
Complete blood count	Hemoglobin (g/dL)	8.2	12.0 - 16.0
	White blood cell count (x10^9^/L)	9.41	4.00 - 10.00
	Platelets (x10^9^/L)	194	140 - 440
	Mean Corpuscular Volume (MCV) (fL)	92.0	78.0 - 98.0
Renal panel	Urea (mmol/L)	14.2	2.5 - 7.8
	Creatinine (µmol/L)	182	45 - 84
Liver function test	Bilirubin (µmol/L)	7	<21
	Alanine transaminase (U/L)	13	6 - 66
	Aspartate transaminase (U/L)	20	12 - 42
Other blood tests	Ferritin (µg/L)	1160	13 - 150
	Transferrin (g/L)	1.1	2.0 - 3.6
	Iron (µmol/L)	15	6 - 35
	Iron saturation (%)	54	20 - 50
	Haptoglobin (mg/dL)	136	30 - 200
	Lactate dehydrogenase (U/L)	294	135 - 350

Further history-taking revealed that she had overdosed on 30 tablets of zopiclone 7.5mg the evening before (about 15 hours ago), as she was having insomnia from a relapse of her depression following her recent diagnosis of SLE. She did not overdose on her other medications. She had also been on mirtazapine 30mg every night (ON) for depression. She was diagnosed as having zopiclone-induced methemoglobinemia, as none of her other medications were deemed to cause methemoglobinemia.

In terms of treatment, her zopiclone was promptly discontinued. She was transfused with 1 pint of packed red blood cells which brought her hemoglobin level up to 9.2 g/dL. As methylene blue could possibly cause serotonin syndrome in patients who are on serotonergic medications, she was treated with IV ascorbic acid 1.5g four times a day (QDS) over two days. Her dyspnea gradually resolved. Her methemoglobin levels also subsequently reduced with improvement in the saturation gap (Table 2).

**Table 2:** Trend of methemoglobin levels and arterial blood gas results before (Day 1) and after treatment.

Investigation	Day 1	Day 2 (Morning)	Day 2 (Evening)	Day 3	Day 4	Normal values
Methemoglobin (%)	10.4	8.9	6.0	1.6	<1.0	<1.0
pH	7.35	7.38	7.38	7.37	7.43	7.35 - 7.45
PaO2 (mmHg)	278	279	252	220	238	83 - 108
PaCO2 (mmHg)	36	31	18	22	23	35 - 45
SaO2 – arterial (%)	100	100	100	100	100	>94
SpO2 – pulse oximeter (%)	91	90	98	98	100	>94

## Discussion

This case highlights several important points in the assessment and management of patients with methemoglobinemia, particularly those on sedative medications for insomnia with a background of antidepressant use.

Clues to watch out for in diagnosing methemoglobinemia

Our patient presented with drowsiness. It may have been tempting to focus on evaluating the drowsiness, but it takes an astute mind to notice the low SpO_2_ levels that did not correspond with the oxygen saturation levels on the blood gas, and which did not improve despite the administration of supplemental oxygen.

Such “refractory hypoxemia” arises due to the fact that pulse oximetry measurements of oxygen saturation (SpO_2_) are based on two different wavelength measurements of 660 nm and 940 nm, the ratio of which allows the distinction between oxyhemoglobin and deoxyhemoglobin. This is then expressed as a percentage of oxygenated hemoglobin, or SpO2. Methemoglobin, however, absorbs light at both of these wavelengths, resulting in an error in estimating the percentage of oxygenated hemoglobin, giving an inaccurate SpO_2 _reading [8].

On the other hand, blood gas measurements of SaO_2_ are calculated from the partial pressure of oxygen in the blood (PaO_2_). In methemoglobinemia, it is falsely elevated as it is based on the assumption that all hemoglobin is either oxyhemoglobin or deoxyhemoglobin [9]. Hence, both the SpO_2_ and SaO_2_ levels are inaccurate in methemoglobinemia, but it is the discrepant values, termed the “saturation gap”, which gives us a clue about the presence of methemoglobinemia [10].

Patients with methemoglobinemia often present with cyanosis and dyspnea. The development of cyanosis correlates with the absolute amount of methemoglobin, and not the percentage of methemoglobin [1]. Our patient’s initial methemoglobin percentage was not high, and coupled with baseline anemia, this corresponded with a low absolute level of methemoglobin, and thus she did not appear cyanotic. However, she was dyspneic due to the reduced oxygen-carrying capacity accorded by her baseline low hemoglobin levels combined with the reduced oxygen delivery to tissues due to her methemoglobinemia. Thus, the symptoms of dyspnea should be taken seriously even if the patient is not cyanotic.

Zopiclone is a rare but possible cause of methemoglobinemia

Zopiclone is a cyclopyrrolone that is structurally dissimilar to benzodiazepines but has similar sedative properties by acting as a modulator of the GABA_A_ receptor. It improves sleep by reducing the time to sleep onset and improving sleep maintenance. They are frequently prescribed for insomnia as they are thought to have fewer side effects compared to benzodiazepines [11]. Zopiclone is now widely prescribed for insomnia, especially among patients with depression [12].

Zopiclone’s side effects of nausea, vomiting, headache, giddiness, lethargy, drowsiness, and altered mental status are well known. Zopiclone-induced methemoglobinemia is much rarer, with only a few case reports identified [13-15]. These case reports indicated methemoglobinemia occurring at doses of 750mg-2250mg of zopiclone ingested, much higher than that seen in our case of 225mg. However, despite the lower levels of methemoglobin, our patient was still symptomatic warranting treatment. Some have reported zopiclone-induced methemoglobinemia with concomitant acute kidney impairment [14] or hemolytic anemia [15]. These were, however, not seen in our patient.

While the exact incidence of methemoglobinemia is not known, a retrospective review of cases of acquired methemoglobinemia in a Hong Kong hospital found that 57% of cases were due to zopiclone overdose [16]. This is concerning because not only prescription trends of zopiclone are increasing, but cases of zopiclone overdose are also on the rise [17]. The exact mechanism of zopiclone-induced methemoglobinemia is not known. Zopiclone undergoes metabolism to 13 different metabolites, mostly through the actions of cytochrome P450-dependent monooxygenase in the liver. It is thought that some of the metabolites that possess amine groups undergo N-hydroxylation to cause oxidation of hemoglobin to methemoglobin, similar to that of benzocaine-induced methemoglobinemia [13].

When standard treatment may not always be applicable in methemoglobinemia

The treatment of methemoglobinemia involves the removal of the inciting agent and the administration of an antidote in symptomatic cases. The antidote of choice is methylene blue, which is reduced by NADPH to leukomethylene blue, which is then capable of reducing methemoglobin to hemoglobin.

However, there may be instances where treatment with methylene blue is not possible. This includes patients with G6PD deficiency, as they lack sufficient NADPH to generate leukomethylene blue. Instead, methylene blue may act as an oxidizing agent to precipitate hemolysis. The other instance where treatment with methylene blue is not possible is when the patient is taking serotonergic medications. Methylene blue is a strong inhibitor of monoamine oxidase, increasing levels of serotonin and causing serotonin syndrome [18].

As such, in our patient on mirtazapine, a noradrenergic and specific serotonergic antidepressant, an alternative form of treatment in the form of ascorbic acid was utilized. Ascorbic acid has a reducing potential as well to convert methemoglobin to hemoglobin. It is usually given intravenously, and doses of up to 10g per dose have been tried [19]. High doses of ascorbic acid may cause increased kidney stone formation, and so should be used cautiously in patients with renal impairment.

No trials have been done to compare methylene blue and ascorbic acid, however the efficacy of methylene blue is generally thought to be better and faster, with improvements in methemoglobin levels seen over several hours, compared to days in cases treated with ascorbic acid [19]. Lastly, blood transfusions and blood exchange may have some utility in treating the symptoms of methemoglobinemia by providing a fresh supply of viable hemoglobin for oxygenation and improving oxygen delivery to the tissues [20].

## Conclusions

Prescriptions of zopiclone are increasing, especially among patients with depression who have insomnia. Other than watching out for the complications of drowsiness and altered mental status in zopiclone overdose, one should also be prudent to watch out for the subtle signs of methemoglobinemia. These patients are often on antidepressant medications, and hence, treatment with ascorbic acid will be required.
